# Antitumour efficacy of VEGFR2 tyrosine kinase inhibitor correlates with expression of VEGF and its receptor VEGFR2 in tumour models

**DOI:** 10.1038/sj.bjc.6602109

**Published:** 2004-08-24

**Authors:** I K Dev, R E Dornsife, T M Hopper, J A Onori, C G Miller, L E Harrington, K M Dold, R J Mullin, J H Johnson, R M Crosby, A T Truesdale, A H Epperly, K W Hinkle, M Cheung, J A Stafford, D K Luttrell, R Kumar

**Affiliations:** 1 GlaxoSmithKline, Five Moore Drive, Research Triangle Park, NC 27709, USA

**Keywords:** angiogenesis, VEGF, VEGFR2, prognostic marker, tyrosine kinase inhibitor

## Abstract

During the development of indazolylpyrimidines as novel and potent inhibitors of vascular endothelial growth factor (VEGF) receptor-2 (VEGFR2) tyrosine kinase, we observed that some human tumour xenografts are more sensitive to VEGFR2 kinase inhibitors than others. A better understanding of the basis for this differential response may help to identify a predictive marker that would greatly aid in the identification of a suitable patient population for treatment. One representative compound from the indazolylpyrimidine series is GW654652 that inhibited all three VEGFRs with similar potency. The inhibition of VEGFR2 kinase by GW654652 was about 150 to >8800 more potent than the inhibition of eight other kinases tested. GW654652 inhibited VEGF- and bFGF-induced proliferation in endothelial cells with an IC_50_ of 110 and 1980 nM, respectively, and has good pharmacokinetic profile in mouse and dog. We investigated the association between VEGF and VEGFR2 expression and the antitumour efficacy of GW654652, in various xenograft models. Statistically significant associations were observed between the antitumour efficacy of GW654652 in xenografts and VEGF protein (*P*=0.005) and VEGFR2 expression (*P*=0.041). The oral dose of GW654652 producing 50% inhibition of tumour growth (ED_50_) decreased with increasing levels of VEGF (*r*=−0.94); and, in contrast, the ED_50_ increased with the increased expression of VEGFR2 (*r*=0.82). These results are consistent with the observed inverse correlation between VEGF and VEGFR2 expression in tumours. These findings support the hypothesis that VEGF and VEGFR2 expression by tumours may predict the therapeutic outcome of VEGFR kinase inhibitors.

Vascular endothelial growth factor (VEGF) and its receptors have been implicated in the angiogenesis that is essential for growth and metastasis of solid tumours. Since formation of solid tumours is angiogenesis dependent, several strategies have been developed to inhibit VEGF signal transduction as part of anticancer therapy (reviewed in McMahon, 2000). These include monoclonal antibodies against VEGF and its receptor, VEGFR2 (vascular endothelial growth factor receptor-2), as well as VEGF-trap that also neutralises VEGF in microcirculation (Kim *et al*, 1993; Holash *et al*, 2002). A recombinant humanised monoclonal version of the anti-VEGF mAb has shown promising results in human cancer patients (Hurwitz *et al*, 2003). An alternate approach to block VEGF signalling is to develop low molecular weight inhibitor of the tyrosine kinase domain of VEGFR2, suitable for chronic oral administration and continual suppression of tumour angiogenesis. Several selective VEGFR2 kinase inhibitors have been developed and have demonstrated efficacy in xenograft models (Fong *et al*, 1999; Wood *et al*, 2000; Laird *et al*, 2002).

VEGFR2 kinase inhibitors are less effective against some tumour xenografts than others (Fong *et al*, 1999; Wood *et al*, 2000; Laird *et al*, 2002). The degree of efficacy of VEGF suppression by an anti-VEGF antibody or a VEGF-Trap also differs markedly in different experimental tumours (Kanai *et al*, 1998; Asano *et al*, 1999; Rowe *et al*, 2000; Kim *et al*, 2002). With some of the earlier VEGFR2 kinase inhibitors, SU5416 and PTK787/ZK222584, slower growing tumours were found to be more inhibited by these agents than faster growing tumours (Fong *et al*, 1999; Wood *et al*, 2000). More recently, Laird *et al* (2002) concluded that differences in growth rates were unlikely to be the key determinants of differential tumour responses to the VEGFR2 kinase inhibitor, SU6668, because all tumour models examined in the study were fast growing *in vivo*. We were interested in a better understanding of the basis for this differential response to various anti-VEGF therapies that may help to identify a predictive marker(s) of response, and would greatly aid in the identification of a suitable patient population for treatment.

The clinical success of Gleevec/STI-571 was greatly facilitated by the presence of the Philadelphia chromosome in chronic myelogenous leukaemia as a diagnostic marker and by the ability to monitor the disease via the analysis of white blood cell counts (Druker and Lydon, 2000). The oestrogen receptor status of primary breast cancers has been shown to predict the benefit of adjuvant tamoxifen in prolonging both disease-free interval and overall survival (reviewed in Jordan, 2000). Similarly, expression of Her2 and CD20 antigen was useful in development of Trastuzumab and Rituxan (McLaughlin *et al*, 1998; Vogel *et al*, 2002). Although several small molecules and protein therapeutics targeting VEGF signalling have shown encouraging clinical results, clinical development of VEGFR-targeted therapies has been more challenging due to lack of a suitable diagnostic marker (Kindler *et al*, 2001; George *et al*, 2003; Hurwitz *et al*, 2003; Kuenen *et al*, 2003; Raymond *et al*, 2003; Yang *et al*, 2003).

Overexpression of VEGF has been shown to correlate with increased risk of metastatic disease and overall poor prognosis in different carcinomas (reviewed in Ferrara, 1999). Elevated VEGF expression has also been used as a marker to select tumour types in some of the early clinical trials with anti-VEGF therapies. Vascular endothelial growth factor is abnormally overexpressed in highly vascular clear-cell renal carcinoma (RCC) due to the deregulation of VEGF degradation resulting from mutations in the VHL gene (Iliopoulos *et al*, 1996). Bevacizumab, a neutralising mAb to VEGF, significantly prolonged time-to-disease progression but failed to show an increase in overall survival in patients with metastatic RCC (Yang *et al*, 2003). Semaxanib/SU5416, a VEGFR2 kinase inhibitor, demonstrated preliminary evidence of activity in RCC and mesothelioma patients (Kindler *et al*, 2001; Kuenen *et al*, 2003); however, it failed to show clinical benefit in large Ph III studies in patients with colorectal cancer. Similarly, SU11248 and PTK787 also showed tumour responses in Ph I studies in RCC (George *et al*, 2003; Raymond *et al*, 2003). A recent Ph III study with bevacizumab in colorectal cancer patients showed improved response rate and overall survival when given with standard chemotherapy (Hurwitz *et al*, 2003). The prognostic value of VEGF and its receptors in relation to anti-VEGF therapies, however, has not been studied as widely.

The present study describes a novel and selective VEGFR2 kinase inhibitor, GW654652 (Kumar *et al*, 2003; Cheung *et al*, 2003) and the association between its antitumour efficacy and the expression of VEGF and VEGFR2 in various xenograft models. We observed that the elevated expression of VEGF in tumour models is associated with the increased sensitivity to GW654652. Moreover, expression of VEGF was inversely related to VEGFR2 expression in tumour xenografts, which is consistent with the published observations that VEGF downregulates VEGFR2 expression by turnover of receptor at the cell surface.

## MATERIALS AND METHODS

### Materials

Human tumour cell lines SW620, HT29, HCT116 (colon carcinoma), A375P (melanoma), and PC3 (prostate adenocarcinoma) were obtained from the American Type Culture Collection (Rockville, MD, USA). HN5, human head & neck carcinoma cells, were kindly provided by Helmout Modjtahedi at the Institute of Cancer Research, Surrey, and UK. Human umbilical vein endothelial cells (HUVEC) were obtained from Clonetics (San Diego, CA, USA). GW654652, *N*^2^-[5-(ethylsulphonyl)-2-methoxyphenyl]-*N*^4^-methyl-*N*^4^-(3-methyl-1*H*-indazol-6-yl)pyrimidine-2,4-diamine; GW612286, *N*^4^-(3-methyl-1*H*-indazol-6-yl)-*N*^2^-(3,4,5-trimethoxyphenyl)pyrimidine-2,4-diamine; and GW695612X, 4-chloro-3-({4-[methyl(3-methyl-1*H*-indazol-6-yl)amino]pyrimidin-2-yl}amino)benzenesulphonamide were synthesised at GlaxoSmithKline (Durham, NC, USA) (Table 1

**Table 1:** Enzyme and cellular potency of VEGFR2 kinase inhibitors

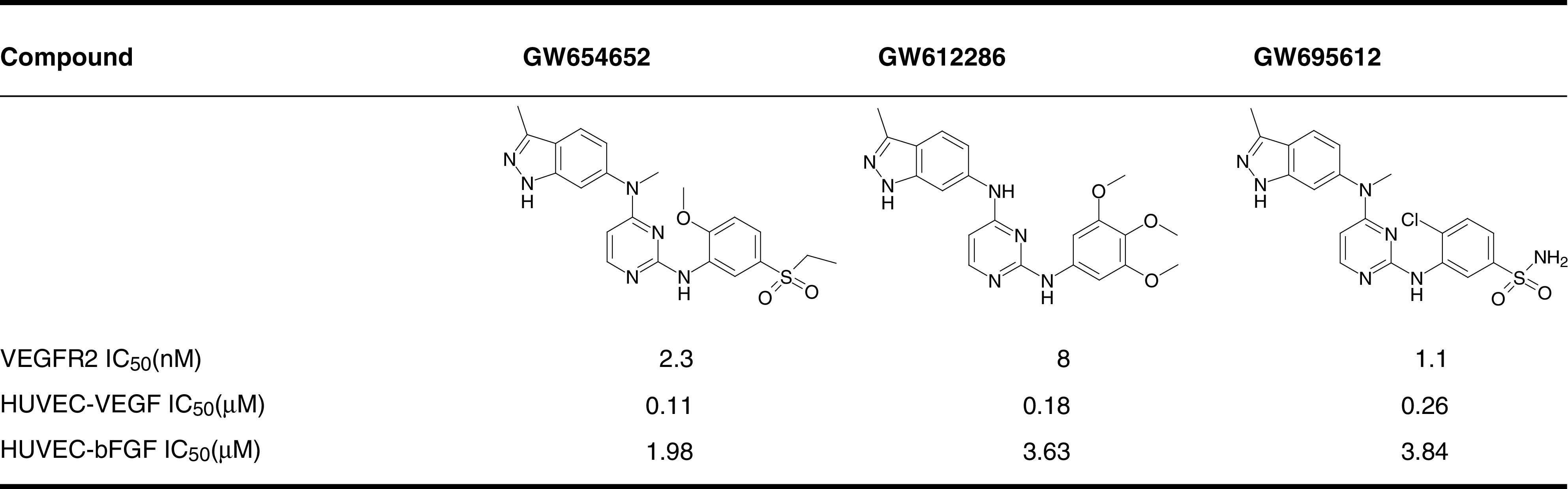

IC_50_ of various small molecules were determined against human VEGFR2 kinase as well as HUVEC grown in presence of VEGF or bFGF as described in Materials and Methods.

HUVEC=human umbilical vein endothelial cells; VEGFR=vascular endothelial growth factor (VEGF) receptor; IC=inhibitory concentration.

). Human and mouse VEGF Elisa kits were obtained from R&D Systems (Minneapolis, MN, USA).

### Kinase assays

Vascular endothelial growth factor receptor kinase assays were carried out in a homogenous time-resolved fluorescence (HTRF) format in 384-well microtitre plates using a purified, baculovirus-expressed GST-fusion protein encoding the catalytic carboxyl-terminus of human VEGFR kinase 1, 2, or 3. Reactions were initiated by the addition of 10 *μ*l of activated VEGFR kinase (1 nM, 0.1 M HEPES pH 7.5, 0.1 mg ml^−1^ BSA, and 0.30 mM DTT) to 10 *μ*l of substrate (360 nM biotin-aminohexyl-EEEEYFELVAKKKK-NH_2_ peptide, 75 *μ*M ATP, 10 mM MgCl_2_) and 1 *μ*l of small molecule inhibitor at various concentrations. Plates were incubated at room temperature for 60 min, and the reaction was stopped by the addition of 20 *μ*l 100 mM EDTA. Homogenous time-resolved fluorescence reagents (20 *μ*l 15 nM streptavidin-linked allophycocyanin, 1 nM europium-labelled anti-phosphotyrosine antibody in 0.1 M HEPES, pH 7.5, 0.1 mg ml^−1^ BSA) were added and plates were incubated for a minimum of 10 min. The fluorescence at 665 nm was measured with a Victor plate reader (Wallac, Shelton, CT, USA) using a time delay of 50 *μ*s. The data for dose responses were plotted as % inhibition calculated with the data reduction formula: 100 × (1−(*U*1−*C*2)/(*C*1−*C*2)) *vs* concentration of compound where *U* is the unknown value, *C*1 is the average control value obtained for 1 *μ*l DMSO, and *C*2 is the average control value obtained for 0.035 M EDTA. Data were fitted with a curve described by: *y*=((*V*_max_ × *x*)/(*K*+*x*)+*Y*2), where *V*_max_ is the upper asymptote, *Y*2 is the *Y* intercept, and *K* is the IC_50_.

Inhibition of several other kinases by small molecules was also determined. Each kinase assay was conducted using purified recombinant catalytic domain of the enzyme. The concentration of ATP and kinase-specific biotinylated peptide in each assay was below the apparent *K*_m_ of the respective substrate. Inhibition of c-Fms, SRC, and Tie-2 was evaluated by an HTRF format and the inhibition of CDK2, CDK4, EGFR, ErbB2, and Eph-B4 was detected by scintillation proximity assay.

### Cellular proliferation assays

Effect of kinase inhibitors on cell proliferation was measured using BrdU incorporation method using commercially available kits (Roche Diagnostics, Indianapolis, IN, USA). Briefly, HUVEC were seeded in a medium containing 5% FBS in type 1 Collagen-coated 96-well plates and incubated overnight at 37°C, 5% CO_2._ The medium was aspirated from the cells, and various concentrations of kinase inhibitors in serum-free medium were added to each well. After 30 min, VEGF (10 ng ml^−1^) or bFGF (0.3 ng ml^−1^) was added to the wells. Cells were incubated for an additional 72 h and BrdU (10 *μ*M) was added during the last 18–24 h of incubation. Data were fitted with a curve described by the equation, *y*=*V*_max_ × (1−(*x*/(*K*+*x*))), where *K* is equal to the IC_50_.

### Tumour xenografts

Tumours were initiated by injection of tumour cell suspension subcutaneously in 8–12-week-old nude mice, except PC3 tumours that were grown in SCID mice (Charles River Laboratories, Wilmington, MA, USA). When tumours reached a volume of 100–200 mm^3^, mice were randomised into groups of eight prior to treatment with VEGFR kinase inhibitors. Animals were treated with kinase inhibitors (10, 30, or 100 mg kg^−1^) or vehicle (0.5% hydroxypropyl methyl cellulose, 0.1% Tween 80 in sterile water), administered once or twice daily by oral gavage for 2–3 weeks (till the mean tumour volume reached 1000–1500 mm^3^). Tumour volume was measured twice weekly by calipers, using the formula (length × width × width × 0.5), where length was the longest diameter across the tumour, and width was the corresponding perpendicular. Tumour growth inhibition was calculated by change in the slopes of tumour growth for control and treated tumours. The oral dose of GW654652 producing 50% inhibition of tumour growth (ED_50_, mg kg^−1^) was estimated by a programme that performed a weighted nonlinear regression analysis of data using the equation: *y*=*V*_max_(1−(*x*/(*K*+*x*))), where *K* is equal to ED_50_. All animal studies were carried out with the appropriate institutional ethical committee approval and they met the standards of both the US federal regulations and those required by the UKCCCR guidelines (Workman *et al*, 1998).

### Staining of tumour cells for flow cytometry

Freshly excised tumours (400–800 mm^3^) were dissociated into single-cell suspension by enzymatic digestion with DNAse and collagenase. Cells were fixed and permeabilised with LeucoPerm (Serotec, Kidlington, UK) and labelled for 30 min at 4°C with anti-FLK-1 antibody (clone A-3, SantaCruz, CA, USA). Cells were then incubated with goat anti-mouse-biotin antibody followed by streptavidin-FITC. Labelled cells were analysed with an FACSort (Becton Dickinson, San Jose, CA, USA) and the percentage of VEGFR2-positive cells and channel differences were determined using CellQuest software.

### Western blot analysis of VEGFR2

Snap-frozen tumour xenograft tissues were lysed in RIPA buffer (150 mM NaCl, 50 mM Tris, 1% NP-40, 0.25%. Na-deoxycholate, 0.1% SDS, 1 mM sodium fluoride, 1 mM fresh sodium orthovanadate, and protease inhibitors). Protein concentrations were determined using a BCA kit, and 120–200 *μ*g of tumour extract was loaded onto a 3–8% Tris-acetate gel. To quantitate the VEGFR2 expressions in lungs, 25 *μ*g of lung extracts (obtained by lysing frozen lung samples from Swiss nude female mice injected with VEGF_121_ (R&D Systems) was loaded onto a 3–8% Tris-acetate gel. Proteins were transferred to nitrocellulose membranes. Membranes were blocked for 1 h in TBS (25 mM Tris-HCl, pH 7.4, 150 mM NaCl, 2.7 mM KCl) containing 5% (w/v) low-fat milk. Membranes were then probed with a VEGFR2-specific anti-Flk1 antibody (crossreacts with both mouse and human VEGFR2; clone A-3, Santa Cruz), followed by a donkey anti-mouse-HRP antibody. ECL (Amersham) was used for detection, and densitometric analysis of receptor expression was carried out using a BioRad Fluor-S MultiImager). To confirm equal protein loading, membranes were stripped and reprobed with antibody against *β*-tubulin (Santa Cruz).

### Vascular endothelial growth factor levels in xenograft tumours

Human and mouse VEGF levels in tumour extracts were determined by an immunoassay, according to the manufacturer's instructions (R&D Systems). Samples were analysed by serial dilution and tests were performed at least in duplicates. Vascular endothelial growth factor levels were normalised relative to the protein concentration of the tumour extracts. Circulating human and mouse VEGF levels in plasma were also determined using the same assay.

### Statistical analysis

Spearman's nonparametric test was used to investigate the association between quantitatively measured VEGFR2 expression, VEGF expression, and the antitumour efficacy of GW654652.

## RESULTS

### Antitumour efficacy

**Figure 1:**
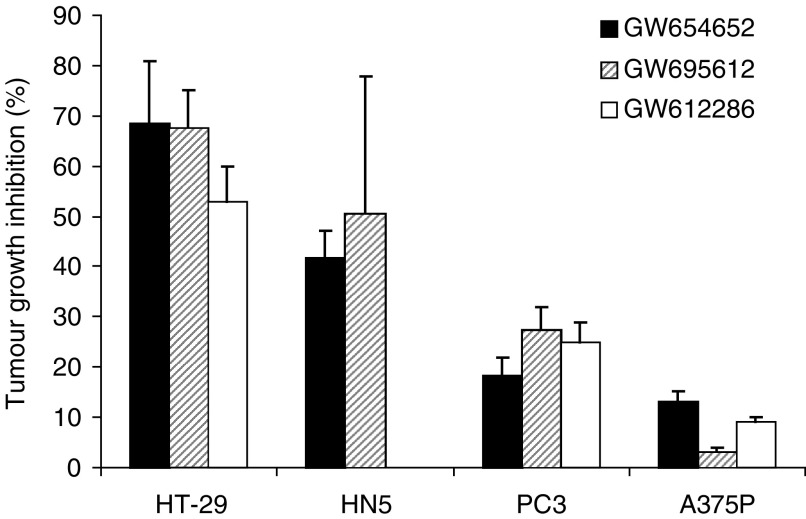
Growth inhibition of human tumour xenografts in mice treated with VEGFR2 kinase inhibitors, GW654652, GW612286, or GW695612. Animals with 100–200 mm^3^ tumour volume were randomly assigned to either vehicle or treatment group (*n*=8 mice group^−1^) as described in Materials and Methods. All compounds were administered orally at 30 mg kg^−1^ once daily, except GW695612 (30 mg kg^−1^, twice day^−1^). Data represent tumour growth inhibition (mean±s.e.m.) in drug-treated animals compared to vehicle-treated mice after 21 days of dosing.

In an effort to identify a suitable VEGFR kinase inhibitor for clinical development, several potent VEGFR2 kinase inhibitors from indazolylpyrimidine series were tested in multiple human tumour xenograft models. Different human tumour xenografts exhibited varying levels of sensitivity to the VEGFR2 kinase inhibitors. Invariably, HN5 and HT29 xenografts showed greater growth inhibition, while A375P and PC3 xenografts demonstrated much lower growth inhibition using multiple compounds at the same dose and schedule. To illustrate this point, antitumour data from three different VEGFR2 kinase inhibitors administered at 30 mg kg^−1^ (orally, once, or twice daily) is shown in Figure 1

. Tumour models HT29 and HN5 were extremely sensitive, as these compounds inhibited tumour growth by 50–70% at this dose. In contrast, PC3 and A375P models were less sensitive (5–20% inhibition) to the VEGFR2 kinase inhibitors at the same dose (Figure 1). Chemical structure, VEGFR2 enzyme activity, and antiproliferative activity against HUVEC growing in presence of VEGF or bFGF for the three compounds are summarised in Table 1.

To further confirm and extend these observations, a more detailed antitumour efficacy study with GW654652 in six different human xenografts was conducted. GW654652 was picked for these studies because of its good pharmacokinetic profile in mouse and dog. GW654652 inhibits all three VEGF receptor kinases with an IC_50_ ranging from 2 to 12 nM (Table 2

**Table 2:** Inhibition of various protein kinases by GW654652

	**IC_50_** **(*μ*M)^a^**	**Fold selectivity *vs* VEGFR-2^b^**
VEGFR-2	0.0023±0.0008	—
VEGFR-3	0.0025±0.0005	1.1
VEGFR-1	0.0120±0.0026	5.3
SRC	0.35±0.24	156
Eph-B4	0.46±0.03	204
c-Fms	0.53±0.33	237
Tie2	0.32±0.04	144
EGFR	1.36±0.42	605
ErbB2	11.53±2.74	5125
CDK2	>20	>8800
CDK4	>20	>8800

a Values are mean±s.e.

b Ratio for the IC_50_ obtained with a given kinase compared to that achieved *vs* VEGFR-2.

VEGFR=vascular endothelial growth factor receptor; IC=inhibitory concentration.

). The inhibition of VEGFR2 kinase by GW654652 was about 150 to >8800 more potent than the inhibition of eight other kinases tested (Table 2). The potent and selective inhibition of VEGFR kinases by GW654652 is also reflected in the potent cellular efficacy against HUVEC stimulated with VEGF compared to bFGF (Table 1). GW654652 has very little effect on the growth of human foreskin fibroblasts or various tumour cell lines in culture (IC_50_ ranging from 4 to >10 *μ*M).

The pharmacokietics and antitumour activity of GW654652 were evaluated in mice at 10, 30 and 100 mg kg^−1^ dose administered orally on a once day^−1^ schedule. An oral dose of 10, 30, and 100 mg kg^−1^ of GW654652 resulted in free *C*_max_ (based on 99% protein binding and total plasma concentration) of 0.06, 0.23, and 1.38 *μ*M, respectively. The plasma concentration remained above the IC_50_ for VEGF-induced HUVEC proliferation for 0, 2, and 12 h for 10, 30, and 100 mg kg^−1^ doses, respectively. GW654652 was a potent inhibitor of the HT29, HCT116 and HN5 xenografts with an ED_50_ ranging from 20 to 28 mg kg^−1^. However, the inhibition of SW620, PC3, and A375P models was less pronounced with an ED_50_ ranging from 46 to 114 mg kg^−1^ dose (Table 3

**Table 3:** Inhibition of tumour growth by GW654652, a VEGFR kinase inhibitor

	**% Inhibition^a^**			
	**10 mg kg^−1^**	**30 mg kg^−1^**	**100 mg kg^−1^**	**ED_50_(mg kg^−1^ day^−1^)**
HT29	5	69	98	20±8
HCT116	18	59	71	21±8
HN5	24	46	83	28±10
SW620	12	44	74	46±11
PC3	ND^b^	21	48	108±50
A375P	ND^b^	14	49	114±54

a % Inhibition of tumour growth compared to vehicle-treated animals.

b Not detectable.

VEGFR=vascular endothelial growth factor receptor.

, Figure 2A

**Figure 2:**
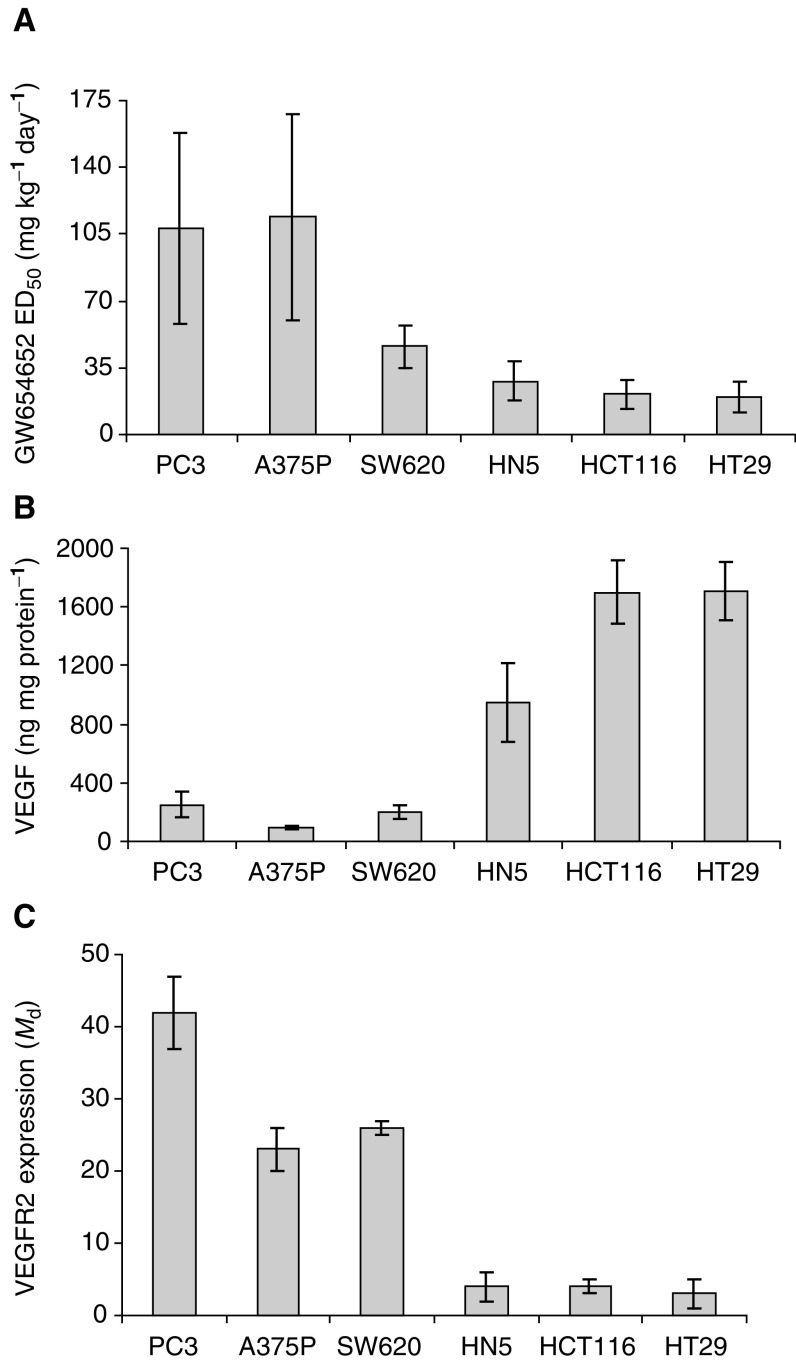
Relationship between (**A**) inhibition of tumour growth by GW654652, (**B**) human VEGF expression, and (**C**) VEGFR2 expression in human tumour xenografts. All values of VEGF levels (ELISA) and VEGFR2 expression (represented as *M*_d_: mean channel difference from FACS analysis) are mean±s.e. and were obtained by analysing 4–15 tumour samples.

).

### Correlation of VEGF expression with antitumour activity of GW654652

The differential response of the tumours to VEGF/VEGFR antagonists may be due to differences in the levels of either the ligand or the receptor in these tumours. Concentrations of mouse and human VEGF in the tumours and in circulation were measured in the human xenografts. Mouse VEGF levels were very similar in different tumour models and probably are not the major determinants of the differential response to VEGFR2 kinase inhibitors. Median circulating mouse VEGF levels ranged from 59 to 94 pg ml^−1^ in plasma of mice with and without various tumour xenografts and the tumour content ranged from 12 to 28 ng mg^−1^ protein in different models. No detectable circulating hVEGF was found (detection limit 4 pg ml^−1^). In contrast, very high and variable levels of hVEGF in different tumour models were observed. The levels of hVEGF varied from 93 ng mg^−1^ of A375P tumour protein to 1710 ng mg^−1^ of HT29 tumour protein (Figure 2B). Relative abundance of hVEGF mRNA correlated well with the protein content of the tumour tissues (data not shown). A comparison of antitumour activity of GW654652 with the hVEGF levels suggested that the oral dose of GW654652 producing 50% inhibition of tumour growth in mice decreased with the increasing concentrations of hVEGF in the tumours (Figures 2A and B). Statistically significant inverse correlation between hVEGF protein expression and the dose of GW654652 that produces 50% inhibition of human tumour xenografts was observed (*r*=−0.94, *P*=0.005).

### Correlation of VEGFR2 expression with antitumour activity of GW654652

**Figure 3:**
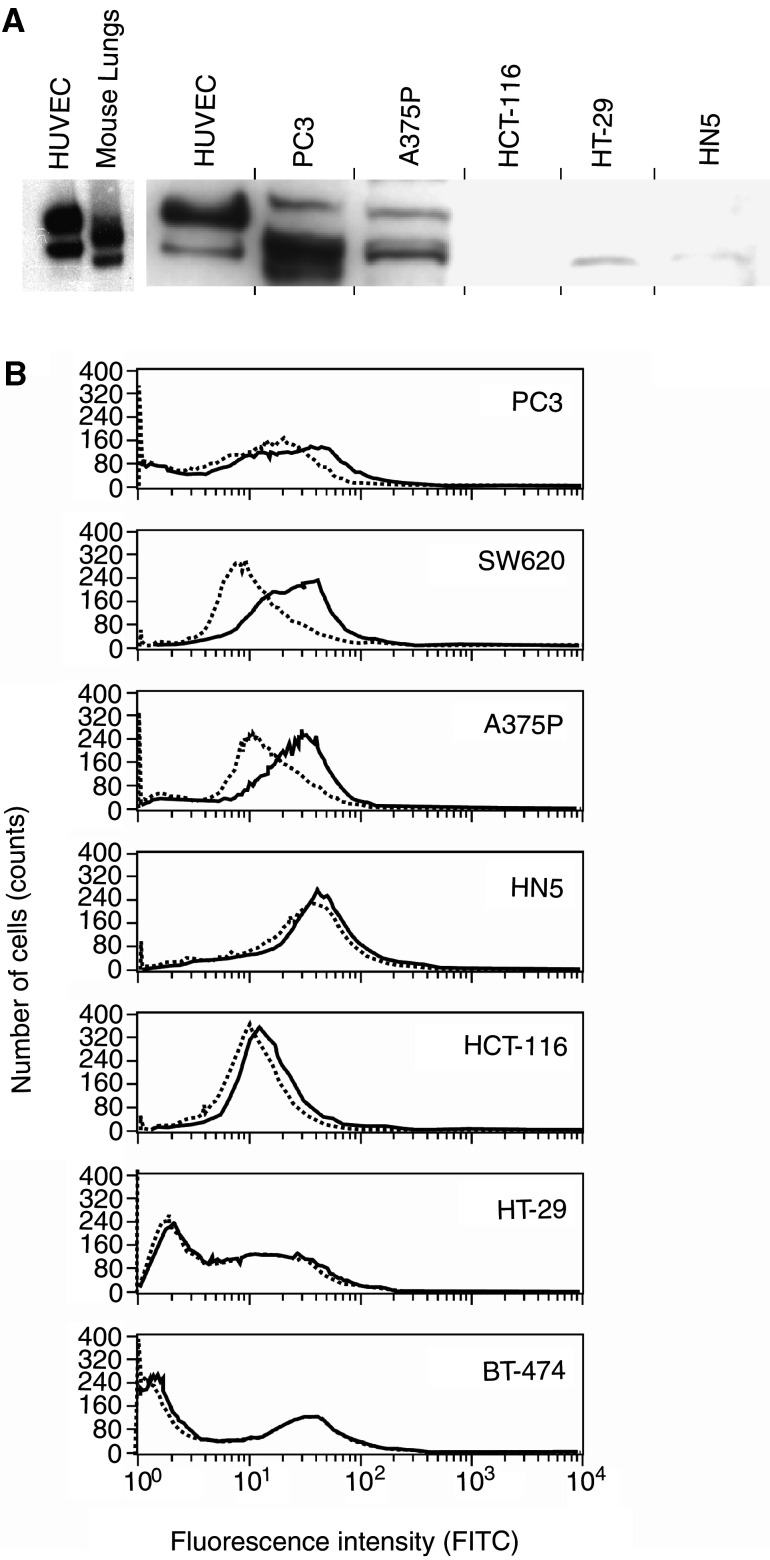
Expression of VEGFR2 by human tumour xenografts. (**A**) Western blot analysis of VEGFR2 in tumour extracts. The amount of protein analysed for PC3=120 *μ*g, A375P=200 *μ*g, HCT116=180 *μ*g, HT29=200 *μ*g, and HN5=200 *μ*g. (**B**) Expression of VEGFR2 by human tumour xenografts. FACS analysis of VEGFR2 expression in PC3, SW620, A375P, HN5, HCT116, and HT29 tumours dissociated as single-cell suspensions. The fluorescence profiles of cells treated with VEGFR2 antibody (solid line) and isotype controls (dotted line).

The VEGFR2 protein expression in tumour xenografts was analysed by flow cytometry using single-cell suspensions from freshly harvested tumour tissue (Ziegler *et al*, 1999). The FACS data for four or five different tumour samples for each xenograft was collected in four-decade mode, gated for single cells, analysed to calculate the mean channel difference (*M*_d_), which represents the magnitude of difference between VEGFR2-specific staining and nonspecific isotype staining. Figure 2C depicts the mean values and s.e.m. for each xenograft. Histograms showing fluorescence intensities of VEGFR2 for a representative tumour sample of each xenograft are shown in Figure 3B

. The VEGFR2 expression was higher in PC3, SW620, and A375P tumours compared to other xenografts tested (Figures 2C and 3B).

In agreement with these FACS results, Western blot analysis of PC3 and A375P tumour extracts also showed readily detectable protein bands of VEGFR2 which were absent or barely detectable in HT-29, HCT-116, and HN5 tumours (Figure 3A). Multiple VEGFR2 protein bands that may correspond to different glycosylated forms of human VEGFR2 expressed by the tumour cells and mouse VEGFR2 expressed by the host endothelial cells were observed in PC3 and A375P tumours. For comparison purposes, human VEGFR2 from HUVEC and the mouse VEGFR2 from mouse lungs analysed on a separate gel are shown in Figure 3A. Although PC3 xenograft express the highest level of VEGFR2 protein followed by A375P melanoma xenograft, the relative intensities of the hVEGFR2 and mVEGFR2 protein bands in PC3 and A375P tumours were not quantified due to the comigration of the two forms on the gel (Figure 3A).

A comparison of antitumour activity of GW654652 with the VEGFR2 expression revealed that the oral dose of GW654652 producing 50% inhibition of tumour growth in mice increased in rank order with the increasing levels of VEGFR2 in the tumours (Figures 2A and C). A statistically significant association was also seen between antitumour activity of GW654652 and VEGFR2 expression by tumour xenografts (*r*=0.82, *P*=0.041).

### Modulation of VEGFR2 by VEGF *in vivo*

**Figure 4:**
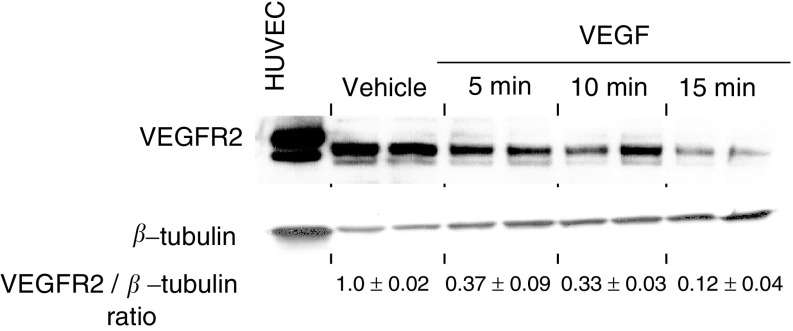
Modulation of VEGFR2 by VEGF *in vivo*. The VEGF_121_ (15 *μ*g/mouse) was administered via tail vein in mice, and their lungs harvested at the indicated time points. Values represent the ratio of VEGFR2 to *β*-tubulin as determined by densitometry and normalised to vehicle-treated mice. Human umbilical vein endothelial cells immunoprecipitated using anti-VEGFR2 antibody from 350 *μ*g protein were used as controls.

A strong inverse correlation between VEGF levels and the VEGFR2 expression among various xenografts was also observed (*r*=−0.85, *P*=0.016; Figures 2B and C). Thus, we examined the effect of VEGF on VEGFR2 expression *in vivo*. Since lung tissue contain high amounts of endothelial cell expressing VEGFR2, we looked at the direct effects of recombinant human VEGF_121_ on the VEGFR2 levels in murine lungs. After the intravenous injection of VEGF_121_ in mice, the lungs were collected after 5, 10, and 15 min, and the VEGFR2 levels were determined by Western blots. The receptor levels decreased as a function of time in mice injected with VEGF_121_ compared to untreated (data not shown) or vehicle-treated animals (Figure 4

). These results are consistent with the earlier observations of Wang *et al* (2000) showing that the cell surface expression of VEGFR2 is regulated by VEGF in cultured endothelial cells.

## DISCUSSION

Patients whose tumours produce high levels of VEGF or other markers of aggressive neovascularisation have significantly poor prognosis with respect to disease progression as well as survival (reviewed in Ferrara, 1999). Increased expression of VEGF in certain carcinomas leads to the development of highly vascularised tumours. Consequently, it has been hypothesised that an inhibitor of VEGF signalling would be particularly efficacious in inhibiting tumour growth in the patients that overexpress VEGF (Mendel *et al*, 2000). However, a clear relationship between increased VEGF expression and the efficacy of anti-VEGF therapy is not yet established. In this study, we present data that show significant associations between the antitumour efficacy of GW654652, a novel and selective VEGFR2 inhibitor, and VEGF protein in xenografts. The tumours that produce higher levels of VEGF were inhibited more effectively by GW654652 and required lower oral doses of the compound to produce 50% inhibition of tumour growth (Figure 2; *r*=−0.94, *P*=0.005). High levels of VEGF expression alone are capable of initiating angiogenesis in a quiescent tumour vasculature; however, many other factors are also involved in angiogenic switch and maturation of tumour vasculature (Bergers and Benjamin, 2003). In agreement with these observations, our data suggest that VEGF is the major determinant of tumour growth but certainly do not rule out the possibility that other factors may also be involved in determining the sensitivity of the tumours to a VEGFR2 inhibitor. Data from the SW620 xenograft that has low VEGF and high VEGFR2 but reasonable sensitivity to the VEGFR2 kinase inhibitor are consistent with this hypothesis (Figure 2).

Interestingly, we also observed that the tumours with increased VEGF expression have lower levels of VEGFR2 expression (Figures 2B and C). Others have observed that under normal physiological conditions VEGF modulates VEGFR2 by downregulating cell surface expression of VEGFR2 in cultured endothelial cells (Wang *et al*, 2000). We examined the effect of VEGF on VEGFR2 expression *in vivo* and demonstrated that the VEGFR2 receptor levels decreased significantly as a function of time in lungs from animals injected with VEGF (Figure 4). An interesting inverse association between VEGF and VEGFR2 and overall survival in CLL patients has also been observed (Aguayo *et al*, 2000; Ferrajoli *et al*, 2001). Our data support the hypothesis that VEGF negatively regulates VEGFR2 expression and the tumours that produce low levels of VEGF show elevated VEGFR2 expression and decreased sensitivity to VEGFR2 kinase inhibitors (Figure 2).

Conversely, others have proposed that high level of VEGF receptor expression by tumour cells can also negatively modulate VEGF signalling by sequestering VEGF, which otherwise will bind to the receptors expressed by vascular endothelial cells (Hiratsuka *et al*, 1998; Kearney *et al*, 2002). Vascular endothelial growth factor receptors are in most cases specifically expressed on vascular endothelial cells, but certain tumour cells also express VEGFR2 (Masood *et al*, 1997; Dias *et al*, 2000; Masood *et al*, 2001; Podar *et al*, 2001; Strizzi *et al*, 2001; Jackson *et al*, 2002; Nakopoulou *et al*, 2002). The expression of VEGFR2 by tumour cells may be a mechanism to become less dependent on VEGF signalling for tumour angiogenesis as well as to negatively regulate the VEGF expression (Hiratsuka *et al*, 1998; Kearney *et al*, 2002). Consistent with this hypothesis, the two tumours PC3 and A375P, which express very high levels of human tumour VEGFR2 as determined by Western blots and mRNA analysis also express very low levels of VEGF (Figure 2 and data not shown).

The reasons why the elevated expression of VEGFR2 in tumours is associated with the decreased sensitivity to VEGFR inhibitors are unknown. However, others have demonstrated that the decreased activity of an anticancer agent could also stem from an increased expression of the target enzyme, in addition to other mechanisms. Elevated expression of thymidylate synthase (TS) protein as a result of gene amplification has been described in tumour cells selected *in vitro* and *in vivo* for drug resistance by exposure to fluoropyrimidine cytotoxic drugs (Berger *et al*, 1985; Clark *et al*, 1987). In fact, TS activity has been associated with response to 5-FU in a number of human cancers, and patients with low TS levels were more likely to respond compared to patients with high TS levels (Kornmann *et al*, 1997; Allegra *et al*, 2003). Similarly, the inefficiency of chemotherapy with the antifolate methotrexate can also stem from an increased expression of dihydrofolate reductase (Alt *et al*, 1978).

Our present results indicate that tumours producing higher levels of VEGF elicit decreased expression of VEGFR2 and increased sensitivity to a VEGFR2 kinase inhibitor. The inverse relationship between the VEGF expression and the VEGFR2 expression is consistent with the hypothesis that VEGF modulates VEGFR2 by downregulating cell surface expression of VEGFR2 in tumours. These results suggest that VEGFR kinase inhibitors may be more effective in patients with tumours expressing high VEGF and low VEGFR2. Future clinical trials should investigate whether expression of VEGF or VEGFR2 in tumour biopsies from patients receiving VEGFR2 kinase inhibitors can be used as diagnostic markers for VEGF-targeted therapies.
